# Viromes outperform total metagenomes in revealing the spatiotemporal patterns of agricultural soil viral communities

**DOI:** 10.1038/s41396-021-00897-y

**Published:** 2021-02-21

**Authors:** Christian Santos-Medellin, Laura A. Zinke, Anneliek M. ter Horst, Danielle L. Gelardi, Sanjai J. Parikh, Joanne B. Emerson

**Affiliations:** 1 Department of Plant Pathology, University of California, Davis, CA USA; 2 Department of Land, Air and Water Resources, University of California, Davis, CA USA; 3 Genome Center, University of California, Davis, CA USA

**Keywords:** Microbial ecology, Soil microbiology, Metagenomics, Metagenomics

## Abstract

Viruses are abundant yet understudied members of soil environments that influence terrestrial biogeochemical cycles. Here, we characterized the dsDNA viral diversity in biochar-amended agricultural soils at the preplanting and harvesting stages of a tomato growing season via paired total metagenomes and viral size fraction metagenomes (viromes). Size fractionation prior to DNA extraction reduced sources of nonviral DNA in viromes, enabling the recovery of a vaster richness of viral populations (vOTUs), greater viral taxonomic diversity, broader range of predicted hosts, and better access to the rare virosphere, relative to total metagenomes, which tended to recover only the most persistent and abundant vOTUs. Of 2961 detected vOTUs, 2684 were recovered exclusively from viromes, while only three were recovered from total metagenomes alone. Both viral and microbial communities differed significantly over time, suggesting a coupled response to rhizosphere recruitment processes and/or nitrogen amendments. Viral communities alone were also structured along an 18 m spatial gradient. Overall, our results highlight the utility of soil viromics and reveal similarities between viral and microbial community dynamics throughout the tomato growing season yet suggest a partial decoupling of the processes driving their spatial distributions, potentially due to differences in dispersal, decay rates, and/or sensitivities to soil heterogeneity.

## Introduction

Viruses are ubiquitous and abundant members of Earth’s ecosystems that can affect the assembly, dynamics, and function of microbial communities [1–3]. They control the size of microbial populations via infection and lysis, redirect microbial metabolism through auxiliary metabolic genes [4–8], and mediate gene transfer across hosts [9, 10]. In soils, 1 g can harbor up to 10^10^ viruses [11, 12], sometimes surpassing the number of coexisting bacteria [13]. Similar to their role in marine systems [3], recent studies suggest that viruses may be key contributors to carbon and nutrient cycling in terrestrial environments [14–16]. Despite this ecological relevance, soil viral communities and the factors shaping them are poorly understood [9, 10, 17].

Viral replication depends on the successful infection of suitable hosts. As such, the abundances of viral populations and, consequently, the structure of viral communities are inherently linked to the compositional trends of coexisting host communities [18–20]. In agricultural soils, rhizosphere processes can alter microbial diversity by actively promoting or inhibiting the recruitment of select taxa [21, 22], and soil amendments can further affect microbiome structure [23]. Understanding whether viral communities display similar trends to those of other microbiota is essential for unraveling the potential of host–virus interactions to affect microbially influenced soil properties. Additionally, environmental factors such as temperature, pH, nutrient status, and moisture could directly contribute to viral community variation by differentially impacting the activity and decay of soil viruses [24]. Similarly, viral adsorption to soil particles could influence the spatial distribution of viruses by limiting virion movement across the soil matrix [24]. Thorough characterization of viral diversity patterns across a variety of soil conditions could shed new light on the drivers of viral community dynamics.

Given the absence of a universal marker gene across viral genomes, metagenomic approaches are necessary to survey viral community diversity [25]. For soils, recent studies have usually relied on the recovery of viral sequences from whole shotgun metagenomic datasets [14, 26, 27]. This approach not only capitalizes on the existence of streamlined wet lab workflows but also facilitates the simultaneous characterization of the microbial and viral community members [10]. While convenient, most sequences in these total metagenomes are derived from bacterial and eukaryotic genomes, presumably concealing the less abundant viral signal unless deep sequencing efforts are performed [10]. Moreover, the vast microbial richness associated with soil environments [28] typically results in high-complexity sequence profiles that are challenging to assemble de novo [29], further hindering the identification of viral genomes.

Viral communities can also be characterized by physically separating virions from larger microbes through filtration prior to DNA extraction and sequencing. The resulting viral size-fraction metagenomes, or viromes, have increased coverage of viral sequences and can therefore capture a more complete picture of viral diversity relative to total metagenomes [10, 30]. While this viromic approach has been successfully adopted to study aquatic systems [3, 31–33], it has been challenging to implement for soil environments [10], mainly due to low DNA extraction yields that can limit library construction and sequencing [30]. Early soil viromic studies used multiple displacement amplification (MDA) and/or random amplified polymorphic DNA (RAPD) PCR to bypass this limitation [34], however, amplification biases, particularly for MDA, preclude quantitative estimations of viral community composition using these approaches [35]. Until recently, large amounts of soil input were required to avoid these amplification biases, but recent improvements in library construction from nanograms of DNA now make it practical to work with manageable amounts of soil (~50 g or less) per sample [30]. Additionally, given that adsorption of viral particles to the soil matrix can limit recovery from soil and passage through filters when removing cellular contamination, the optimization of elution buffers that disrupt the interactions between virions and soil particles is necessary to minimize biases in viromic profiles [10]. Continuous development of extraction workflows [13, 36] has recently enabled virome preparation from a variety of soils [15, 37], greatly expanding our ability to examine viral diversity in these environments.

Here, we use a combination of total metagenomes and viromes to characterize soil dsDNA viral communities associated with an agricultural tomato field. First, we compare the performance of the two profiling approaches, in terms of their ability to recover diverse viral sequences, and then we explore viral and microbial ecological patterns in our data. We find that viromes vastly outperform total metagenomes in the recovery of viral diversity and that viral communities display strong spatiotemporal dynamics, which are only partially explainable by shared patterns with bacterial host communities and environmental conditions.

## Materials and methods

### Sample collection

Samples were collected from a tomato agricultural field within the context of a larger ongoing study of the impacts of biochar, a carbon-rich soil amendment produced by thermal decomposition of organic material [38], on agricultural production. The field, located in Davis, CA, USA (38°32′08″N, 121°46′22″W), consisted of 34 m^2^ of flat land with no discernible slopes. In order to homogenize the soil and minimize legacy effects, the field was tilled (west to east and south to north) in the fall of 2017. Shortly thereafter, the field was divided into three blocks (61 m × 18.4 m), each with forty experimental plots (6.1 m × 4.6 m) arranged in a 10-by-4 layout. Each plot had three 1.5-m-wide beds with lengthwise subsurface drip tape installed in the center. We sampled eight plots from the westernmost block, each treated with one of four biochar treatments (650 °C pyrolyzed coconut shell [Cool Planet, Greenwood Village, CO, USA], 650 °C pyrolyzed pine feedstock [Cool Planet], 800 °C pyrolyzed almond shell [Premier Mushrooms and Community Power Corporation, Colusa, CA, USA], and no biochar, Supplementary Table 1) and one of two nitrogen fertilization regimes (150 or 225 lbs N/acre) (Supplementary Fig. 1A). For all sampled plots, 4.2 kg of biochar (dry weight equivalent) were applied via subsurface banding across each bed on November 8, 2017. Briefly, biochar was spread from buckets by hand into open trenches (30 cm deep) directly above preinstalled drip tape. Biochar-filled trenches were immediately closed, burying the concentrated biochar at the center of each bed. This approach allows biochar to be placed within the rooting zone of plants while minimizing wind erosion risk [39]. Tomato seedlings (cultivar H-8504) were transplanted on May 2, 2018, and nitrogen fertilizer was fed through subsurface drip irrigation on five occasions from May 31 to July 24, 2018 (Supplementary Fig. 1B). Soil samples from the same eight plots were collected on April 23 (at the preplanting stage and before any nitrogen additions) and August 28, 2018 (at the tomato ripening stage) for a total of 16 samples.

Soils were harvested using 2.5-cm-diameter probes to collect the 0–30 cm depth range, which corresponds to the approximate depth of tillage. To avoid the installed drip tape, a composite of eight soil probes was collected within 1-m-long transects parallel to and 15 cm away from the center of the plot, with four probes on each side. Given that biochar amendments were directly applied on top of the drip tape, the presence of biochar particles in the collected soils was minimal. In August, tomato root systems were present throughout each plot, so samples were rhizosphere-influenced. Composite soil samples were stored in sterile plastic bags and transported to the laboratory on ice. Within 48 h, soil samples were sieved to 8 mm and divided for chemistry, moisture, viromics, and total metagenomics. For each time point, samples were processed by one person in a single batch, and it was the same person for both batches.

### Soil chemistry and moisture

The soil is classified as Yolo silt loam, a fine-silty, mixed, superactive, nonacid, thermic Mollic Xerofluvent [40]. Gravimetric moisture content was measured as previously reported [41]. Total nitrogen and carbon (combustion method) and extractable ammonium and nitrate (flow injection analysis on 2 M KCl soil extracts) were measured by the University of California Davis Analytical Lab (Davis, CA, USA). Soil pH (1:1 soil:water), organic matter (loss on ignition), phosphorus (weak Bray and sodium bicarbonate-P), and extractable cations (potassium, magnesium, calcium, and sodium) were measured by A&L Western Labs (Modesto, CA, USA).

### DNA extractions

A detailed description is provided in the Supplementary material. Briefly, for viromics, viral size fractionation was achieved by resuspending 50 g of soil in 70 ml amended potassium citrate prime (AKC’) buffer [42], followed by filtration through a 0.22 µm membrane. Ultracentrifugation was used to concentrate purified virus-like particles, and DNase treatment was used to remove free DNA prior to virion lysis and DNA extraction with the PowerSoil kit (Qiagen, Hilden, Germany). Total DNA for metagenomics was extracted from 0.5 g of soil with the PowerSoil kit.

### Library construction and DNA sequencing

Library construction and high-throughput sequencing were performed by the DNA Technologies and Expression Analysis Core at the UC Davis Genome Center. Libraries for April samples were prepared with the DNA Hyper Prep library kit (Kapa Biosystems-Roche, Basel, Switzerland) and libraries for August samples were prepared with the Nextera DNA Flex Library kit (Illumina, San Diego, CA) (Supplementary Fig. 1C). We were not aware of the differences in library construction methods between sample sets until they became obvious during our bioinformatic processing of the data. Paired-end sequencing (150 bp) was performed across two lanes of the Illumina HiSeq 4000 platform (Illumina), one for each collection time point: viromes and total metagenomes were pooled at equimolar ratios for April samples, and at a 1:2 ratio for August samples. The difference in pooling ratios was deliberate in order to increase the sequencing throughput from the August total metagenomes. All raw sequences have been deposited in the NCBI Sequence Read Archive under the BioProject accession PRJNA646773.

### Read processing and data analysis

A detailed description is provided in the Supplementary material. Briefly, quality filtering was performed with Trimmomatic [43] and BBDUK [44], followed by de novo assembly with MEGAHIT [45] and clustering with PSI-CD-HIT [46]. VirSorter [47] and DeepVirFinder [48] were used to detect viral contigs, and vConTACT2 [49] was used to assign taxonomic classifications. Read mapping was performed with BBMap [44], and viral operational taxonomic units (vOTU) coverage tables were generated with BamM [50]. Thresholds for defining (≥10 Kbp, ≥95% global identity) and detecting (≥75% of the contig length covered ≥1x by reads recruited at ≥90% average nucleotide identity) viral populations (vOTUs) were implemented in accordance with benchmarking and community consensus recommendations [51, 52]. Detection and classification of 16S rRNA gene fragments were performed with SortMeRNA [53] and the RDP classifier [54]. K-mer profiling was performed with sourmash [55, 56]. All statistical analyses were done in R using the vegan [57] and DESeq2 [58] packages. All scripts and intermediate files are available at github.com/cmsantosm/SpatioTemporalViromes/.

## Results and discussion

### Viromes outperform total metagenomes in the recovery of viral sequences from complex soil communities

**Fig. 1:**
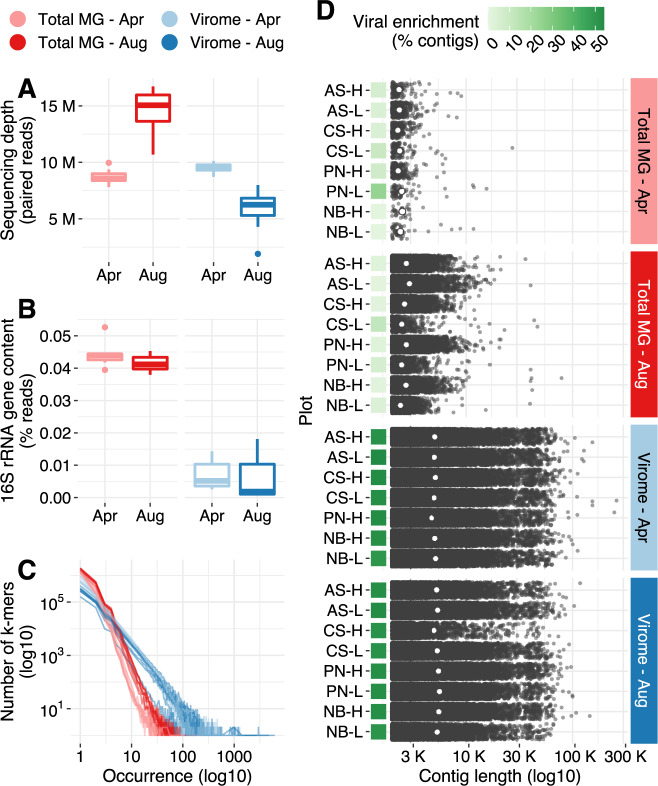
Differences in sequence composition and assembly performance between total metagenomes and viromes. **A** Sequencing depth distribution across profiling methods and time points. The *y*-axis displays the number of paired reads in each library after quality trimming and adapter removal. Boxes display the median and interquartile range (IQR), and data points further than 1.5x IQR from box hinges are plotted as outliers. **B** Percent of reads classified as 16S rRNA gene fragments in the set of quality trimmed reads; the distribution of data within boxes, whiskers, and outliers is as in **A**. **C** Sequence complexity as measured by the frequency distribution of a representative set of k-mers (*k* = 31) detected in each library. The *x*-axis displays occurrence, i.e., the number of times a particular k-mer was found in a library, while the *y*-axis shows the number of k-mers that exhibited a specific occurrence. **D** Length distribution of contigs assembled from each library (min. length = 2Kbp). White dots represent the N50 of each assembly, and green squares display the viral enrichment, as measured by the percent of contigs classified as putatively viral by DeepVirFinder and/or VirSorter. Total MG = total metagenome.

To determine the extent to which viral sequences were enriched and bacterial and archaeal sequences were depleted in viromes, relative to total metagenomes, we performed a series of analyses to compare these two approaches. After quality filtering, total metagenomes yielded an average of 8,741,015 paired reads per library for April samples and 14,551,631 paired reads for August samples, while viromes yielded an average of 9,519,518 and 5,770,419 paired reads in April and August, respectively (Fig. 1A and Supplementary Table 2). Viromes displayed a significant depletion of bacterial and archaeal sequences, as evidenced by fewer reads classified as 16S rRNA gene fragments: 0.006% of virome reads, compared to 0.042% of reads in total metagenomes (Fig. 1B). Moreover, taxonomic classification of the recovered 16S rRNA gene reads revealed clear differences in the microbial profiles associated with each approach: total metagenomes were significantly enriched in Acidobacteria, Actinobacteria, Firmicutes, and Thaumarchaeota, whereas viromes were significantly enriched in Armatimonadetes, Saccharibacteria, and Parcubacteria (Supplementary Fig. 2A, B). These last two taxa belong to the candidate phyla radiation and are typified by small cells [59–61], which would be more likely to pass through the 0.22-um filter that we used for viral particle purification [37, 62]. Although we acknowledge that taxon-specific differences in 16S rRNA gene copy numbers could theoretically account for some of the observed differences in absolute numbers of reads assigned to 16S rRNA genes between viromes and total metagenomes [63], in the context of subsequent analyses (see below), the most parsimonious interpretation is that both the abundances and types of bacterial and archaeal genomic content differed between the two datasets.

To assess differences in sequence complexity between the two profiling methods, we calculated the k-mer frequency spectrum for each library (Fig. 1C). Relative to viromes, total metagenomes displayed an increased number of singletons (k-mers observed only once) and an overall tendency toward lower k-mer occurrences, indicating that size-fractionating our soil communities reduced sequence complexity. These differences in sequence complexity translated into notable contrasts in the quality of de novo assemblies obtained from individual libraries (Fig. 1D), while viromes yielded 800 Mbp of assembled sequences across 169,421 contigs (250 Mbp assembled in ≥10 Kbp contigs), total metagenomes produced only 65 Mbp across 22,951 contigs (1.5 Mbp assembled in ≥10 Kbp contigs). The improved assembly quality from the viromes was despite lower sequencing throughput relative to total metagenomes, particularly for the August samples (Fig. 1A). Using DeepVirFinder [48] and VirSorter [47] to mine assemblies for viral contigs, we found that 52.4% of virome contigs and only 2.2% of total metagenome contigs were identified as viral. Together, these results show that our laboratory methods for removing contamination from cells and free DNA reduced genomic signatures from cellular organisms, substantially improved sequence assembly, and successfully enriched the viral signal in soil viromes relative to total metagenomes.

### Viromes facilitate exploration of the rare virosphere

To remove redundancy in our assemblies, we clustered all 192,372 contigs into a set of 105,909 representative contigs (global identity threshold = 0.95). Following current standards to define viral populations (vOTUs) [51, 52], we then screened all nonredundant ≥10 Kbp contigs for viral signatures. We identified 4065 vOTUs with a median sequence length of 17,870 bp (max = 259,025 bp) and a median gene content of 27 predicted ORFs (max = 421 ORFs). To profile the viral communities in our samples, we mapped reads against this database of nonredundant vOTU sequences (≥90% average nucleotide identity, ≥75% coverage over the length of the contig). On average, 0.04% of total metagenomic reads and 23.4% of viromic reads were mapped to vOTUs (Supplementary Fig. 3A). One August virome sample (CS-H) had particularly low sequencing throughput and low vOTU recovery (Fig. 1A and Supplementary Fig. 3B) and was discarded from downstream analyses.

**Fig. 2:**
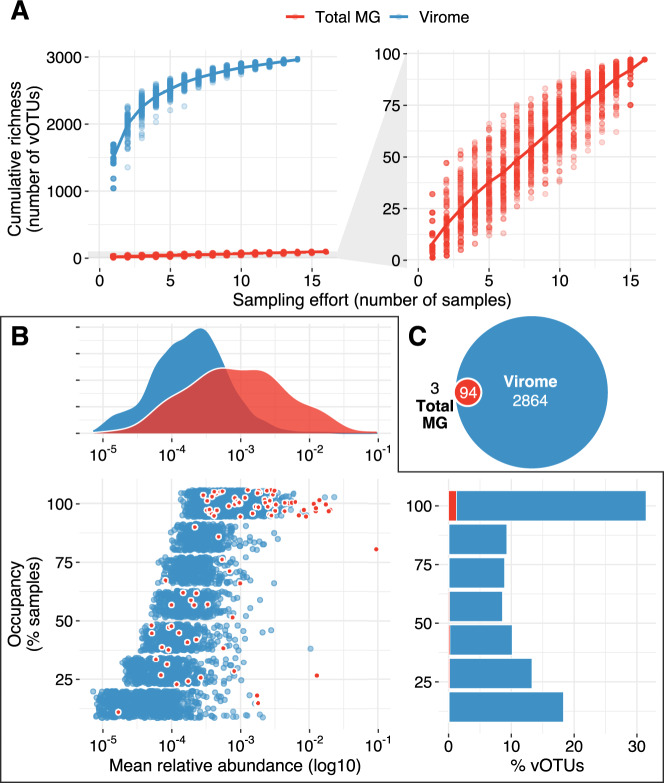
Viral richness, abundance, and occupancy patterns captured by viromes compared to total metagenomes. **A** Accumulation curves of vOTUs in total metagenomes (red, *n* = 16) and viromes (blue, *n* = 14). Dots represent cumulative richness at each sampling effort across 100 permutations of sample order; the overlaid line displays the mean cumulative richness. The right graph includes the same total metagenomic data as the left graph, zoomed in along the *y*-axis. **B** Abundance-occupancy data based on vOTU profiles derived from viromes. Data in blue are from vOTUs detected only in viromes, and data in red are from vOTUs detected in both viromes and total metagenomes. Bottom left: dots represent the mean relative abundance (*x*-axis) and occupancy (percent of samples in which a given vOTU was detected, *y*-axis) that individual vOTUs displayed in viromes within a collection time point (April or August). Thus, vOTUs detected in both time points are represented twice. Red dots highlight the set of vOTUs shared between total metagenomes and viromes. Top: density curves showing the distribution of relative abundances for all vOTUs detected in viromes (blue) or the subset of vOTUs detected in viromes and total metagenomes (red). Bottom right: percent of vOTUs (*x*-axis) found at each occupancy level (*y*-axis). Red bars highlight the percent of vOTUs detected in both profiling methods. **C** Euler diagram displaying the overlap in detection for each vOTU (*n* = 2961) across profiling methods. Red vOTUs were detected by both profiling methods, and three vOTUs were detected exclusively in total metagenomes. Total MG = total metagenome.

In total, 2961 vOTUs were detected through read mapping in at least one sample. Of these, 2864 were exclusively found in viromes, 94 in both viromes and total metagenomes, and three in total metagenomes alone. Thus, viromes were able to recover 30 times as many viral populations as total metagenomes, even when vOTUs assembled from viromes were part of the reference set for read mapping. Notably, the three vOTUs exclusively detected in total metagenomes were only present in one metagenome from April that did not have a successful paired virome (Supplementary Fig. 4). Considering that all other vOTUs detected in total metagenomes were detected in at least one virome, it seems possible that the corresponding virome could have contained these vOTUs if sequencing had been successful. Consistent with capturing a representative amount of viral diversity from the viromes but not total metagenomes, our sampling effort was sufficient to approach a richness asymptote in vOTU accumulation curves derived from viromes but not total metagenomes (Fig. 2A).

To examine the distribution of vOTUs along the abundance-occupancy spectrum, we compared mean relative abundances of vOTUs against the number of samples in which each vOTU was detected. Given the contrasting experimental conditions between the April and August collections, we performed this analysis within each time point. In viromes, highly abundant vOTUs tended to be recovered in the majority of samples (i.e., they displayed high occupancies), while rare vOTUs were typically recovered in only a few samples (Fig. 2B, C and Supplementary Fig. 5A), a trend usually observed in microbial communities [64]. Furthermore, more than 30% of vOTUs were found in all sampled plots, indicating the presence of a sizable core virosphere distributed throughout the field. In contrast, the distribution of 16S rRNA gene OTUs in viromes leaned toward lower occupancies (Supplementary Fig. 5B) as expected from the significant depletion of cellular genomes upon size fractionation (Fig. 1B). On the other hand, more than 80% of vOTUs in total metagenomes were detected only once (Supplementary Fig. 5C), despite the widespread distribution displayed by the 16S rRNA gene OTUs identified in the same samples (Supplementary Fig. 5D), suggesting a sparse recovery of viral diversity compared to a more complete recovery of bacterial and archaeal diversity in total metagenomes.

Inspecting the abundance-occupancy patterns for the 94 vOTUs detected in both viromes and total metagenomes revealed that vOTUs recovered from total metagenomes were among the most abundant and ubiquitous in virome profiles (Fig. 2B), indicating that total soil metagenomes were more likely to miss the rare virosphere. Notably, comparing the relative abundances of vOTUs across paired total metagenomes and viromes showed that their abundance-based ranks were not always preserved (Supplementary Fig. 4). While this discrepancy could stem from methodological challenges associated with virome preparations (e.g., differential adsorption of viruses to the soil matrix could have impacted their resuspension and recovery, therefore affecting the relative abundances of the associated vOTUs), it is also likely that total metagenomes were more susceptible to subsampling biases as evidenced by the sparse and inconsistent recovery of vOTUs exhibited by this profiling method (Supplementary Fig. 5C).

### Viromes reveal a diverse taxonomic landscape

**Fig. 3:**
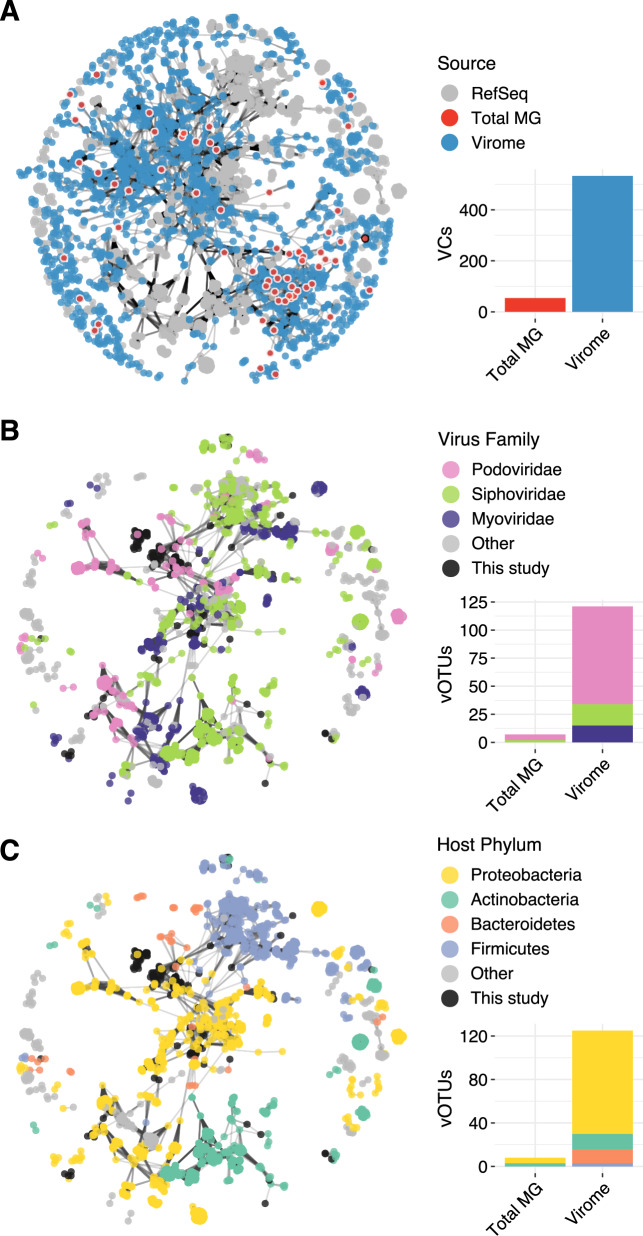
Taxonomic diversity and predicted hosts of viral populations (vOTUs) identified in viromes compared to total metagenomes. **A** Gene-sharing network of vOTUs detected in viromes alone (blue nodes), total metagenomes (red nodes; nodes outlined in white were also detected in viromes, nodes outlined in black were detected exclusively in total metagenomes), and RefSeq prokaryotic virus genomes (gray nodes). Edges connect contigs or genomes with a significant overlap in predicted protein content. Only vOTUs and genomes assigned to a viral cluster (VC) are shown. Accompanying bar plots indicate the number of distinct VCs detected in total metagenomes and viromes (VCs detected in both profiling methods are counted twice, once per bar plot). **B**, **C** Subnetwork of all RefSeq genomes and co-clustered vOTUs. Colored nodes indicate the virus family (**B**) or the associated host phylum (**C**) of each RefSeq genome. Bar plots display the number of vOTUs classified as each predicted family (**B**) or host phylum (**C**) across total metagenomes and viromes (vOTUs detected in both profiling methods are counted twice, once per bar plot). Total MG = total metagenome.

To examine the taxonomic spread covered by our vOTUs, we compared them against the RefSeq prokaryotic virus database using vConTACT2, a network-based method to classify viral contigs [49]. Under this approach, vOTUs are grouped by shared predicted protein content into taxonomically informative viral clusters (VCs) that approximate viral genera. Of the 2961 vOTUs, 1712 were confidently assigned to VCs, while the rest were only weakly connected to other clusters (outliers, 784 vOTUs) or shared no genus-level predicted protein content with any other contigs (singletons, 465 vOTUs) (Supplementary Table 3). Only 130 vOTUs were grouped with RefSeq genomes, indicating that this dataset has substantially expanded known viral taxonomic diversity (Fig. 3A). Subsetting the vOTUs detected by each profiling method showed that viromes captured a more taxonomically diverse set of viruses: 1711 vOTUs detected in viromes were assigned to 533 VCs, while 68 vOTUs detected in total metagenomes (67 of which were also detected in viromes) were assigned to 54 VCs (53 of which were also detected in viromes). Thus, any potential biases in the types of viruses recovered through soil viromics (e.g., through preferential recovery of certain viral taxonomic groups from the soil matrix) were not immediately obvious. Any such biases were either eclipsed by the much greater taxonomic diversity of viruses recovered in viromes, relative to total metagenomes, and/or they also apply to total metagenomes, at least for the viruses and soils examined here.

Most of the 130 vOTUs clustered with RefSeq viral genomes could be taxonomically classified at the family level (Fig. 3B). Podoviridae was the most highly represented family, followed by Siphoviridae and Myoviridae. Myoviridae were only detected in viromes, not total metagenomes, further confirming that viromes do not seem to exclude viral groups relative to total metagenomes—if anything, the opposite seems to be true. Among the Siphoviridae clusters, we could further identify three vOTUs as belonging to the genus Decurrovirus, which are phages of *Arthrobacter*, a genus of Actinobacteria common in soil [65–67]. Because the genome network was highly structured by host taxonomy (Fig. 3A, [68]), we used consistent host signatures among RefSeq viruses in the same VC to assign putative hosts to vOTUs in VCs shared with RefSeq genomes. Most such vOTUs were putatively assigned to Proteobacteria, Actinobacteria, or Bacteroidetes hosts, and a few were linked to Firmicutes. Interestingly, these bacterial phyla were among the most abundant taxa in the 16S rRNA gene profiles derived from the total metagenomes from these soils (Supplementary Fig. 2A).

Although soil viromes and total metagenomes have been compared [62] and their presumed advantages and disadvantages have been reviewed [10], here a comprehensive comparison of results from both profiling approaches applied to the same samples showed that soil viromes recover richer (Fig. 2A) and more taxonomically diverse (Fig. 3) soil viral communities than total metagenomes.

### Compositional patterns of agricultural soil viral communities and their ecological drivers

Since viromes vastly outperformed total metagenomes in capturing the viral diversity in our samples, we focused on viromes to explore the compositional relationships among viral communities. To assess the impact of each individual experimental factor on beta diversity, we performed separate permutational multivariate analyses of variance (PERMANOVA) on Bray–Curtis dissimilarities (Supplementary Table 4). Collection time point had a significant effect (*R*^2^ = 0.50, *p* = 0.001), but biochar (*R*^2^ = 0.19, *p* = 0.58) and nitrogen (*R*^2^ = 0.12, *p* = 0.75) treatments did not (only samples from the August time points, after nitrogen amendments, were considered for the nitrogen analysis). Additionally, to determine if the location of each sampled plot had an impact on community composition, we tested the effect of plot position along the West–East (W–E) and South–North (S–N) axes of the field (Supplementary Table 4). Viral communities displayed a significant spatial gradient along the W–E axis (*R*^2^ = 0.17, *p* = 0.046) but not the S–N axis (*R*^2^ = 0.10, *p* = 0.20). Given the significant spatiotemporal structuring in our samples, we performed an additional PERMANOVA to examine the effect of biochar while accounting for these factors (Supplementary Table 5) and detected a significant effect (*R*^2^ = 0.19, *p* = 0.012) only when both collection time point and W–E gradient were part of the model. We did not detect a significant effect of nitrogen treatment, even after accounting for the W–E gradient in the August samples (Supplementary Table 5).

To assess whether the bacterial and archaeal communities displayed similar compositional trends and could therefore potentially explain patterns in viral community composition, we attempted to generate metagenome assembled genomes (MAGs) from our total metagenomes. However, the low quality of total metagenomic assemblies (Fig. 1D) precluded MAG reconstruction (19 MAGs with a median completeness of 30.3), so instead we used 16S rRNA gene profiles recovered from total metagenomes (Supplementary Fig. 2A). Although 16S rRNA genes accounted for <0.05% of the reads in our total metagenomes (Fig. 1B), 573 bacterial and archaeal 16S rRNA gene OTUs were detected, and accumulation curves approached richness asymptotes (Supplementary Fig. 2C), suggesting that enough microbial diversity was recovered to justify further analyses. PERMANOVA on Bray–Curtis dissimilarities revealed that only collection time point significantly correlated with bacterial and archaeal community composition, while spatial location, biochar treatment, and nitrogen fertilizer concentration did not (Supplementary Tables 4 and 5).

### Viral and microbial communities display coupled temporal dynamics

**Fig. 4:**
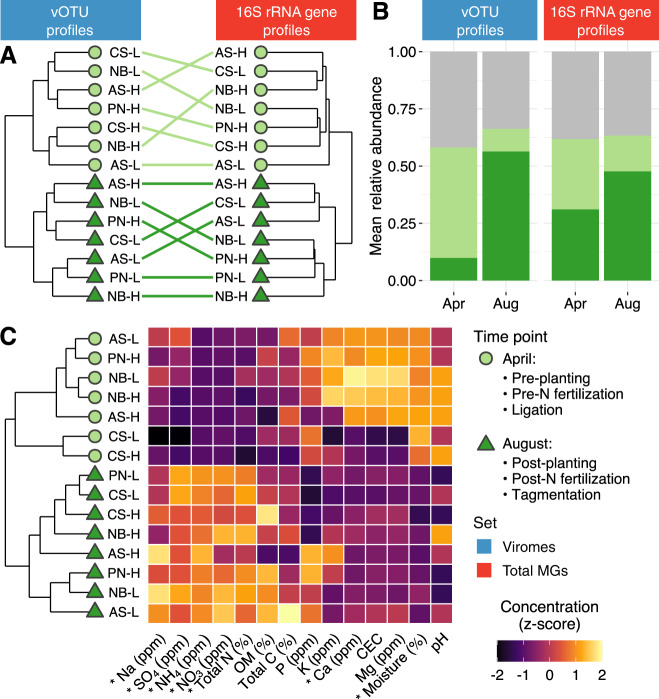
Temporal shifts in viral and microbial communities and soil properties during a tomato growing season. **A** Tanglegram depicting the hierarchical clustering of viral communities derived from viromes (left) and bacterial and archaeal (hereafter, microbial) communities derived from total metagenomes (right) based on Bray–Curtis dissimilarities calculated on Hellinger-transformed relative abundances. Shapes and colors indicate collection time point (legend to the right of **C**). Colored lines connect viral and microbial communities from the same sample. **B** Summed mean relative abundances of the set of vOTUs and 16S rRNA gene OTUs significantly affected by collection time point. Color indicates enrichment in April (light green), August (dark green), or no significant enrichment by time point (gray). **C** Hierarchical clustering of samples based on soil chemical profiles and environmental characteristics. The heatmap shows the *z*-transformation of each measurement across samples. OM = organic matter, CEC = cation exchange capacity, Total MGs = total metagenomes.

Compositional differences between April and August samples were significant for both viral and microbial communities (Fig. 4A), and a Mantel test revealed that the Bray–Curtis dissimilarity matrices of viral and microbial communities were significantly correlated (*r* = 0.59, *p* = 0.0003). Unsurprisingly, due to the reliance of viruses on their hosts for replication, viral and microbial communities have been previously observed to correlate in soil [14, 18], and results here suggest that viruses and their bacterial and archaeal hosts had coupled temporal responses to the same variables and/or to each other.

To identify individual vOTUs that displayed temporal dynamics, we performed a differential abundance analysis between collection time points. Of 2958 vOTUs detected in viromes, 1062 were enriched in April samples, and 681 were enriched in August samples (Supplementary Table 6). The summed relative abundances of these vOTUs accounted for up to 63.3% of the viral communities (Fig. 4B). No clear taxonomic trend could be detected, as very few (92 of 1743) differentially abundant vOTUs could be taxonomically classified, and all identified viral families and putative host phyla were proportionally represented across April- and August-enriched vOTUs (Supplementary Fig. 6A, B).

To identify microbial taxa associated with compositional shifts between time points, we performed a similar differential abundance analysis on the 16S rRNA gene OTU profiles. Of 573 taxa, 38 were enriched in April, and 20 were enriched in August (Supplementary Table 7). This relatively small number of 16S rRNA gene OTUs accounted for 66.3% of the total microbial community abundance (Fig. 4B). Taxa enriched in April encompassed a diverse set of microorganisms, with members of Bacteroidetes, Alphaproteobacteria, Gammaproteobacteria, and Acidobacteria being the most prevalent, while taxa enriched in August were exclusively members of the Actinobacteria or Alphaproteobacteria (Supplementary Fig. 6C). Interestingly, a recent study on greenhouse-grown tomatoes [69] showed similar taxonomic enrichment and depletion patterns in rhizosphere communities relative to bulk soil communities, including enrichment for Actinobacteria and Alphaproteobacteria in the rhizosphere, suggesting that root microbiome recruitment could explain some of the compositional changes between preplanting and harvest time points. These results are consistent with many previous studies that have demonstrated rhizosphere impacts on the diversity and composition of microbial communities [70–73] and, more recently, on RNA viral communities in Mediterranean grasslands [16].

We also inspected soil properties and nutrient profiles to determine whether they followed the same temporal patterns as viral and microbial communities. Missing colon. Indeed, they did: samples grouped into two distinct clusters based on overall chemical composition, one with all April samples and one with all August samples (Fig. 4C). Testing the impact of collection time point on individual chemical measurements revealed that, relative to April soils, August soils exhibited a significant increase in ammonium, nitrate, total nitrogen, sodium, and sulfate concentrations and a significant decrease in soil moisture and calcium content (ANOVA, Supplementary Table 8). Nitrogen amendments, which were applied between sampling time points, have been shown to shift the composition of microbial communities associated with tomato rhizospheres toward an Actinobacteria-enriched state [23], suggesting that the increased abundance of this phylum in August samples may be related to an increase in nitrogen availability, in addition to the presence of tomato roots. Furthermore, a recent survey of rice paddies revealed that nitrogen amendments influenced the absolute abundances of viruses and bacteria [74], indicating that a coupled response by viral and microbial communities could have been triggered by agricultural nitrogen inputs. Considering that soil moisture availability is one of the main factors influencing the composition of soil bacterial communities [75] and that soil desiccation has been linked to viral inactivation [24], it is likely that soil moisture also contributed to viral and bacterial community differences between time points. Similar correlations between viral community composition and soil moisture content have been observed in thawing permafrost peatlands [14].

Altogether, these results indicate that viral and microbial communities displayed strong temporal dynamics that were consistent with responses to changes in both biotic (plant) and abiotic characteristics of soils. Still, we acknowledge that the magnitude of this temporal shift could have been amplified by biases associated with library preparation: while April samples were constructed using a standard ligation-based approach, August samples were inadvertently constructed with a tagmentation protocol due to changes in methodology at the sequencing facility (see “Methods”). Given that nonrandom transposition might skew the compositions of viromic [76] and metagenomic [77] profiles, it is possible that some of the detected temporal differences in our study could stem from technical artifacts. However, considering the consistency of some of our results with previous studies (e.g. specific taxonomic response patterns associated with rhizosphere assembly and nitrogen fertilization), it is likely that, overall, the observed compositional differences represent true ecological patterns over time.

### Viral communities but not microbial communities were spatially structured in the West–East direction across an agricultural field

**Fig. 5:**
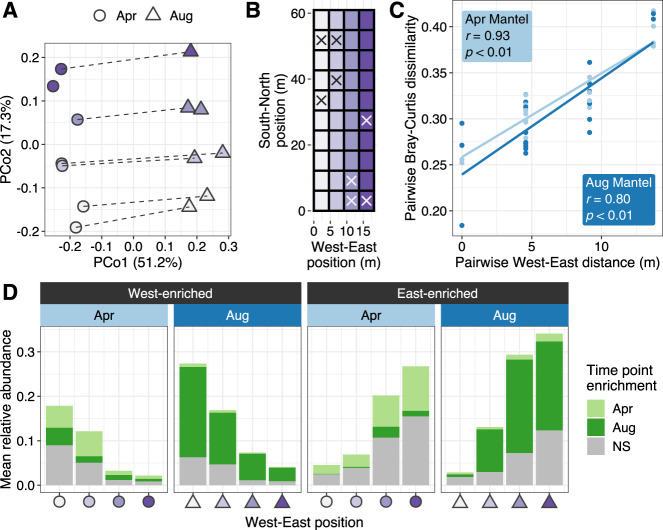
Spatial structuring of viral communities along an 18 m gradient. **A** Unconstrained analysis of principal coordinates based on vOTU Bray–Curtis dissimilarities calculated on Hellinger-transformed relative abundances across virome samples. Shapes represent the collection time point, and colors indicate the position of each plot along the West–East axis of the sampled field, as displayed in **B**. Lines connect April and August samples collected from the same plot. **B** Diagram depicting the spatial distribution of plots. Sampled plots are indicated by an “X”. **C** Correlations between spatial distance across the West–East axis (in meters between plots) and Bray–Curtis dissimilarities of viral communities profiled in viromes. Inset values display the *r* statistic and associated *p* value of Mantel tests performed within each collection time point. The fitted line is provided for visualization purposes only. **D** Shifts in the mean summed relative abundances of significantly West-enriched or East-enriched vOTUs along the W–E axis in April or August. Bar colors indicate whether vOTUs were also significantly enriched in a particular time point (NS = not significant).

To visualize the effect of plot position on the overall structure of viral communities, we performed a principal coordinate analysis on Bray–Curtis dissimilarities calculated across the virome-derived vOTU profiles. While the first axis captured the differences between collection time points, the second axis revealed a compositional transition from the westernmost (W) to the easternmost (E) end of the field (Fig. 5A, B). As expected from the PERMANOVA results (Supplementary Tables 4 and 5), the unconstrained ordination did not display any discernible patterns based on biochar treatment or nitrogen fertilization regime (Supplementary Fig. 7A, B). Comparing the pairwise Bray–Curtis dissimilarities in viral community composition against the corresponding pairwise W–E field distances further confirmed a significant correlation between viral community composition and W–E position (Fig. 5C). This pattern was not recapitulated along the South–North direction. We found 1035 vOTUs displaying a W–E gradient in their relative abundances across the field: 460 with increasing abundances from east to west and 575 with increasing abundances from west to east (Fig. 5D and Supplementary Table 9). Furthermore, examining the overlap with vOTUs affected by collection time point (Fig. 4B) revealed that more than half of the W–E spatially structured vOTUs were temporally dynamic (Supplementary Table 9 and Fig. 5D), suggesting that this W–E structuring of viral communities occurred on time scales captured by this study. In other words, the vOTUs patterned by a W–E gradient in April were generally distinct from those exhibiting similar W–E abundance trends in August, presumably indicating either repeated responses to the same underlying variables and/or repeated patterns of dispersal over time.

We next explored whether this W–E gradient was observed in the bacterial and archaeal communities. As expected from the PERMANOVA results (Supplementary Tables 4 and 5), there was no significant correlation between microbial community composition and spatial distance across the W–E field axis (Supplementary Fig. 8A, B). Thus, while the overarching temporal differences in viral and microbial communities were related, the W–E spatial distribution of viral and microbial communities would seem to have resulted from at least partially decoupled assembly processes. This result is consistent with Mantel tests between viral and microbial community composition, which were significant overall (i.e., driven by temporal separation, see above), but were not significant within each collection time point (April: *r* = 0.12, *p* = 0.27; August: *r* = 0.27, *p* = 0.15). We also analyzed the soil nutrient profiles and did not detect a significant effect of W–E position on any of the measured chemical or environmental properties (Supplementary Table 8).

Given the strong spatiotemporal dynamics displayed by viral communities, we examined the effect of biochar treatment on overall viral community composition through a partial canonical analysis of principal coordinates (CAP) that removed the variance due to collection time point and W–E position. This approach revealed that biochar-amended soils clustered separately from untreated soils and that viral communities associated with different types of biochar were compositionally distinct, a pattern driven by 43 differentially abundant vOTUs (Supplementary Fig. 9A, B and Supplementary Table 10). Similar trends were observed in the 16S rRNA gene profiles of total metagenomes despite the effect of biochar on overall microbial community composition being not significant (Supplementary Fig. 9C and Supplementary Tables 4 and 5). While previous studies have detected clear compositional shifts in the bacterial communities of agricultural fields upon biochar additions [78–80], no consistent pattern has emerged among responsive taxa across different biochar amendments and soil types [81]. Furthermore, while biochar was spread evenly throughout the field in these previous studies, the subsurface banding approach followed in this study constrained the biochar to a narrow area along each bed (Supplementary Fig. 1A). This could explain why none of the measured chemical soil properties was significantly influenced by biochar treatment (Supplementary Table 8), as soil samples were collected 15 cm away from the biochar application zone (Supplementary Fig. 1A). Overall, these results suggest that biochar amendments may have impacted both viral and microbial community composition but that temporal differences (and, for viral communities, W–E spatial differences) were far more pronounced. Thus, future studies that seek to investigate the impacts of soil amendments on viral communities may benefit from rigorous spatiotemporal replication in the study design.

While spatial differentiation has been previously reported for soil viral communities, prior studies considered larger areas with contrasting soil properties. A RAPD DNA study of soil viral diversity along a 4 km land-use transect (including forest, pasture, and cropland systems) found significant differences in viral community composition along the transect [82]. Similarly, a metagenomic characterization of peatland soils found distinct viral communities in three areas comprising different habitats along a permafrost gradient within a ~12,500 m^2^ area [14]. Although the spatial patterns in viral community composition detected in these studies correlated with differences in various environmental parameters, none of the soil properties that we measured in this study were able to explain the observed W–E spatial gradient. Moreover, we found no evidence of W–E spatial structuring in the coexisting bacterial and archaeal communities, suggesting that viruses were uniquely affected by an unknown underlying factor.

One possible explanation for the observed W–E spatial structuring of viral communities could be unidentified legacy effects from previous growing seasons that may have created an unrecognized gradient across the agricultural field (though we note that the field was tilled in both S–N and W–E directions shortly before biochar addition, likely dampening any potential legacy effects, and the field was fallow for at least one year prior to the start of our study). It has been proposed that rates of viral decay might be much slower than viral production in some soils [83], such that an accumulation of older viruses might explain differences in the W–E spatial structuring of viral, relative to microbial communities. However, if this were the case, we would expect most of the W–E spatially structured vOTUs to persist over time and exhibit the same abundance patterns in the April and August samples, yet more than half of these vOTUs were also differentially abundant between time points. Another potential factor that could have contributed to this W–E spatial differentiation is dispersal, consistent with the location of the sampled field next to a gravel road (Supplementary Fig. 1A). We speculate that vehicular traffic could have transported dust from the road to the field and/or provided vibrations to induce shifts in soil pore structure and hydraulic connectivity, both of which would presumably have been more pronounced closer to the source (the road). Additionally, contrasting conditions at the W–E borders of the field (a road on one side and more tomato fields on the other, Supplementary Fig. 1A) could also have led to edge effects. While little is known about the impact of edge effects on soil viruses, other biotic and abiotic properties of agricultural soils have been shown to be influenced by their proximity to the field border [84, 85]. Future studies could be designed to better disentangle the complex interactions of production, decay, selection, and dispersal on soil viral and microbial community composition.

## Conclusions

By comparing total metagenomes and viromes from the same tomato soil samples, we showed that viromes recovered a richer and more taxonomically diverse set of dsDNA vOTUs than total metagenomes, even at lower sequencing depths. Moreover, total metagenomes mostly detected the highly persistent and abundant vOTUs, a pattern that highlights the greater ability of viromes to explore the rare virosphere. Analyzing the beta diversity trends of viral and microbial communities revealed coupled temporal shifts that coincided with changes in the biotic and abiotic properties of soil. Viral communities further displayed a compositional gradient along the sampled agricultural field that was not observed in the microbial communities, suggesting the presence of underlying factors that can differentially affect the spatial distribution of viral and microbial populations. Biochar amendments were also statistically correlated with and thus predicted to impact viral community composition. However, this effect was only evident after accounting for the variation associated with the spatiotemporal patterns structuring viral diversity, emphasizing the importance of replicated experimental design over space and time when evaluating the effect of treatments on soil viral communities in future experiments.

## Supplementary information

Supplemental Material

Supplemental Tables
