# Supplementary material for: Viromes outperform total metagenomes in revealing the spatiotemporal patterns of agricultural soil viral communities
Source: ISME J. 2021 Feb 21;15(7):1956–70. doi: 10.1038/s41396-021-00897-y (PMC8245658; doi:10.1038/s41396-021-00897-y)
Supplement: Supplementary file 1 — Supplemental Material [file 41396_2021_897_MOESM1_ESM.pdf]

Supplementary Material

Supplementary Methods

Soil viral DNA extraction

Viral DNA was extracted from 50 g of fresh soil per sample using a previously reported protocol [1] with minor modifications. For each sample, two 50 mL conical tubes were filled with 25 g of soil and 37.5 mL of 0.02 µm filtered AKC' extraction buffer (per liter: 10% PBS, 10g K Citrate, 1.44 g Na_2HPO_4, 0.24g KH_2PO_4, and 36.97g $MgSO_4$). Resulting slurries were vortexed briefly until homogenized, shaken on an orbital shaker for 15 minutes at 400 RPM, vortexed for 3 minutes, and centrifuged at 4,700 x g for 15 minutes. Supernatant was filtered through a 0.22 µm filter to remove most cells, and supernatants from tubes corresponding to the same sample were combined into one 70 mL polycarbonate ultracentrifuge tube. Supernatants were centrifuged using an Optima LE-80K ultracentrifuge (Beckman-Coulter Life Sciences, Indianapolis, IN, USA) with a 45 Ti rotor at 32,000 x g for 3 hours at 4 °C.

Supernatant was decanted and the resulting pellets (the viral fraction), were resuspended in 200 µl of ultrapure water. Free DNA was removed from the resuspended pellets by treatment with 30 units of RQ1 RNase-free DNase and 30 µl of 10X DNase buffer (Promega Corp., Madison, WI, USA) for two hours at room temperature (22 °C). The reaction was quenched with 30 µl of DNase stop solution (Promega Corp., Madison, WI, USA).

DNA was extracted from the viral fraction using the DNeasy PowerSoil kit (Qiagen, Hilden, Germany), following the manufacturer's protocol, except the bead-beating step was replaced by a 10-minute 70 °C incubation, a 5-second vortex, and a 5-minute 70 °C incubation. Extracted DNA was quantified by an Invitrogen Qubit 4 Fluorometer using a 1x High Sensitivity DNA assay (Thermo Fisher Scientific, Inc., Waltham, MA, USA).

Soil total DNA extraction

Total DNA from soil was extracted from 0.5 g of soil using the DNeasy PowerSoil kit (Qiagen, Hilden, Germany), following the manufacturer's instructions. Extracted DNA was quantified by an Invitrogen Qubit 4 Fluorometer using a 1x High Sensitivity DNA assay (Thermo Fisher Scientific, Inc., Waltham, MA, USA).

Read processing and assembly

Trimmomatic [2] was used to remove library adapters and quality-trim raw reads (minimum q-score of 30 evaluated on 4-base sliding windows; minimum read length of 50). BBDuk [3] was then used to remove PhiX sequences. *De novo* assembly on individual libraries was performed with MEGAHIT [4] in meta-large mode with a contig cutoff size of 2,000 bp. Assembly statistics were generated using the BBTools stats.sh script [3]. To remove redundant contigs across assemblies, we used the PSI-CD-HIT [5] implementation of BLASTN to cluster contigs at a global identity threshold of 0.95.

Detection and classification of viral contigs

We used VirSorter [6] and DeepVirFinder [7] to identify putative viral contigs. For VirSorter, only contigs assigned to categories with the most confident (categories 1 and 4) or likely (categories 2 and 5) predictions were retained; for DeepVirFinder, only sequences with a score ≥ 0.9 and p-value < 0.05 were retained. The union of contigs identified by both methods was used for downstream analyses. Viral contig identification was first performed on individual assemblies to measure the enrichment of viral signal in the set of contigs found in each library. Viral contig identification was also performed on the subset of clustered contigs with lengths ≥ 10 Kbp to generate a database of non-redundant sequences representing viral operational taxonomic units (vOTUs). The length threshold and 95% sequence identity from prior clustering were based on previous benchmarking and definitions of vOTUs [8, 9].

To assign taxonomic classifications to the recovered vOTUs, we first predicted protein content using Prodigal in metagenome mode [10]. The generated amino acid file was then used to build a gene-sharing network using vConTACT2[11] with the following parameters: NCBI RefSeq database of bacterial and archaeal viral genomes (v85) was used as a reference, Diamond [12] was used to calculate protein alignment, and MCL [13] and ClusterOne [14] algorithms were used to calculate protein and genome clusters, respectively.

Read mapping

Read mapping against the database of non-redundant vOTUs was performed with BBMap [3] at a minimum sequence identity of 90%. Resulting SAM files were converted to BAM files and indexed using SAMtools [15]. The parse function of BamM [16] was then used to generate two vOTU tables: one displaying the trimmed pileup coverage (tpmean mode) and the other one displaying the absolute number of mapped reads (counts mode). Finally, we calculated the per-sample horizontal coverage for each vOTU using the genomecov function in BEDtools [17]. We then identified instances in which vOTUs displayed < 75% coverage over the length of the contig and filtered them out of the vOTU tables using an in-house R script (github.com/cmsantosm/SpatioTemporalViromes/blob/master/Processing/Scripts/votu_filtering.Rmd)

Detection and classification of 16S rRNA gene fragments

Reads containing 16S rRNA gene sequences were recovered using SortMeRNA [18] by comparing quality-filtered reads against representative versions of the bacterial and archaeal SILVA databases [19]. The RDP classifier [20] was then used to assign taxonomy. The output hierarchical file was further parsed using the hier2phyloseq function implemented in the RDPutils package [21] to generate a count table.

K-mer profiling

Sourmash [22, 23] was used to compute k-mer signatures for each library using a compression ratio of 1,000 and k-mer size of 31.

Data analysis and visualization

All statistical analyses were conducted using R version 3.6.3. Unless otherwise stated, analyses were performed using the trimmed pileup coverage vOTU table. The vegan package [24] was used for the following analyses: accumulation curves were calculated using the specaccum function, Bray-Curtis dissimilarity matrices were calculated on Hellinger-transformed relative abundances using the vegdist function, permutational multivariate analyses of variance (PERMANOVA) were performed with the adonis function, and Mantel tests were performed with the mantel function. We performed two sets of PERMANOVA: one set testing the individual effects of single factors (**STable 4**) and another set testing the effect of either biochar or nitrogen treatments while accounting for differences due to collection time point and/or spatial structuring along the W-E gradient (**STable 5)**. In the PERMANOVA models, W-E and S-N positions were coded as continuous variables. The function pcoa from the package ape [25] was used to perform principal coordinate analyses. Hierarchical clustering on z-transformed values was performed with the hclust function. Differential abundance analyses were performed with DESeq2 [26] using count tables as input. The factors included in the design formula were those that showed a significant effect in the overall composition analyses: collection time point, W-E gradient, and biochar treatment to model vOTU abundances; and collection time point to model 16S OTU abundances. All plots were generated with the ggplot2 [27], ggdendro [28], GGally [29], and eulerr [30] packages. All scripts and intermediate files are available at github.com/cmsantosm/SpatioTemporalViromes/

References

1. Trubl G, Solonenko N, Chittick L, Solonenko SA, Rich VI, Sullivan MB. Optimization of viral resuspension methods for carbon-rich soils along a permafrost thaw gradient. *PeerJ* 2016; **4**: e1999.
2. Bolger AM, Lohse M, Usadel B. Trimmomatic: a flexible trimmer for Illumina sequence data. *Bioinformatics* 2014; **30**: 2114–2120.
3. Bushnell B. BBTools software package. *URL http://sourceforge net/projects/bbmap* 2014.
4. Li D, Liu C-M, Luo R, Sadakane K, Lam T-W. MEGAHIT: an ultra-fast single-node solution for large and complex metagenomics assembly via succinct de Bruijn graph. *Bioinformatics* 2015; **31**: 1674–1676.
5. Huang Y, Niu B, Gao Y, Fu L, Li W. CD-HIT Suite: a web server for clustering and comparing biological sequences. *Bioinformatics* 2010; **26**: 680–682.
6. Roux S, Enault F, Hurwitz BL, Sullivan MB. VirSorter: mining viral signal from microbial genomic data. *PeerJ* 2015; **3**: e985.
7. Ren J, Song K, Deng C, Ahlgren NA, Fuhrman JA, Li Y, et al. Identifying viruses from metagenomic data using deep learning. *Quantitative Biology* 2020; **8**: 64–77.
8. Roux S, Emerson JB, Eloe-Fadrosh EA, Sullivan MB. Benchmarking viromics: an in silico evaluation of metagenome-enabled estimates of viral community composition and diversity. *PeerJ* 2017; **5**: e3817.
9. Roux S, Adriaenssens EM, Dutilh BE, Koonin EV, Kropinski AM, Krupovic M, et al. Minimum Information about an Uncultivated Virus Genome (MIUViG). *Nat Biotechnol* 2019; **37**: 29–37.
10. Hyatt D, Chen G-L, Locascio PF, Land ML, Larimer FW, Hauser LJ. Prodigal: prokaryotic gene recognition and translation initiation site identification. *BMC Bioinformatics* 2010; **11**: 119.

11. Bin Jang H, Bolduc B, Zablocki O, Kuhn JH, Roux S, Adriaenssens EM, et al. Taxonomic assignment of uncultivated prokaryotic virus genomes is enabled by gene-sharing networks. *Nat Biotechnol* 2019; **37**: 632–639.
12. Buchfink B, Xie C, Huson DH. Fast and sensitive protein alignment using DIAMOND. *Nat Methods* 2015; **12**: 59–60.
13. van Dongen SM. Graph clustering by flow simulation. 2000.
14. Nepusz T, Yu H, Paccanaro A. Detecting overlapping protein complexes in protein-protein interaction networks. *Nat Methods* 2012; **9**: 471–472.
15. Li H, Handsaker B, Wysoker A, Fennell T, Ruan J, Homer N, et al. The Sequence Alignment/Map format and SAMtools. *Bioinformatics* 2009; **25**: 2078–2079.
16. Woodcroft B, Lamberton T, Imelfort M. BamM. 2018.
17. Quinlan AR, Hall IM. BEDTools: a flexible suite of utilities for comparing genomic features. *Bioinformatics* 2010; **26**: 841–842.
18. Kopylova E, Noé L, Touzet H. SortMeRNA: fast and accurate filtering of ribosomal RNAs in metatranscriptomic data. *Bioinformatics* 2012; **28**: 3211–3217.
19. Quast C, Pruesse E, Yilmaz P, Gerken J, Schweer T, Yarza P, et al. The SILVA ribosomal RNA gene database project: improved data processing and web-based tools. *Nucleic Acids Res* 2013; **41**: D590–6.
20. Wang Q, Garrity GM, Tiedje JM, Cole JR. Naive Bayesian classifier for rapid assignment of rRNA sequences into the new bacterial taxonomy. *Appl Environ Microbiol* 2007; **73**: 5261–5267.
21. Quensen J. RDPutils: R Utilities for Processing RDPTool Output. 2018.
22. Pierce NT, Irber L, Reiter T, Brooks P, Brown CT. Large-scale sequence comparisons with *sourmash*. *F1000Res* 2019; **8**: 1006.
23. Titus Brown C, Irber L. sourmash: a library for MinHash sketching of DNA. *JOSS* 2016; **1**: 27.

24. Oksanen J, Blanchet FG, Friendly M, Kindt R, Legendre P, McGlinn D, et al. vegan: Community Ecology Package. 2018.

25. Paradis E, Claude J, Strimmer K. APE: Analyses of Phylogenetics and Evolution in R language. *Bioinformatics* 2004; **20**: 289–290.

26. Love MI, Huber W, Anders S. Moderated estimation of fold change and dispersion for RNA-seq data with DESeq2. *Genome Biol* 2014; **15**: 550.

27. Wickham H. ggplot2: Elegant Graphics for Data Analysis. 2016. Springer-Verlag New York.

28. de Vries A, Ripley BD. ggdendro: Create Dendrograms and Tree Diagrams Using 'ggplot2'. 2016.

29. Schloerke B, Crowley J, Cook D, Briatte F, Marbach M, Thoen E, et al. GGally: Extension to 'ggplot2'. 2018.

30. Larsson J. eulerr: Area-Proportional Euler and Venn Diagrams with Ellipses. 2020.

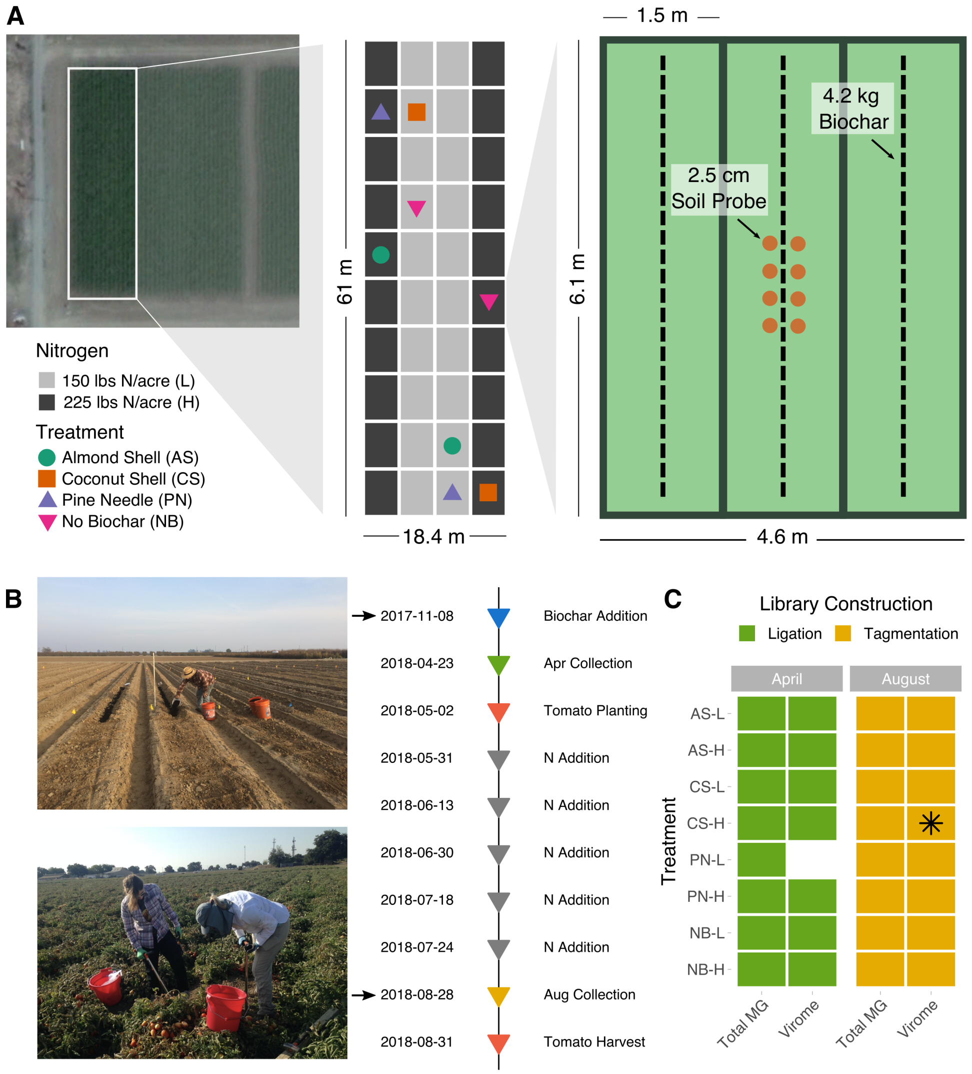

Supplementary Figure 1

(A) Left panel: aerial view of the agricultural field; the highlighted area indicates the experimental block used for this study. Picture was taken on July 10th, 2018, in the middle of the tomato growing season (see B). Middle panel: diagram depicting the spatial distribution of plots within the block: dots indicate the eight sampled plots with color and shape corresponding to the biochar treatment; nitrogen fertilization treatment is indicated by the color of the individual plots (low - light gray, high - dark gray). Fertilization began between the two collection time points (see B). Right panel: diagram depicting an individual plot. Dotted black lines represent the approximate area where biochar was buried along each bed. Brown dots indicate the approximate location of the eight soil cores harvested for each sample. (B) Pictures on the left show how biochar additions (up) and August sample collection (down) were performed. Right: timeline of the growing season, biochar and nitrogen amendments, and collection time points. Note that the distance between time points is not to scale. (C) Library construction workflows used to generate the total metagenomes (MG) and viromes. The blank space represents a virome sample that failed at the library construction step, and the asterisk highlights the virome sample omitted from compositional analyses due to suboptimal sequencing throughput and read mapping (see Figures 1A and D and Supplementary Figure 3).

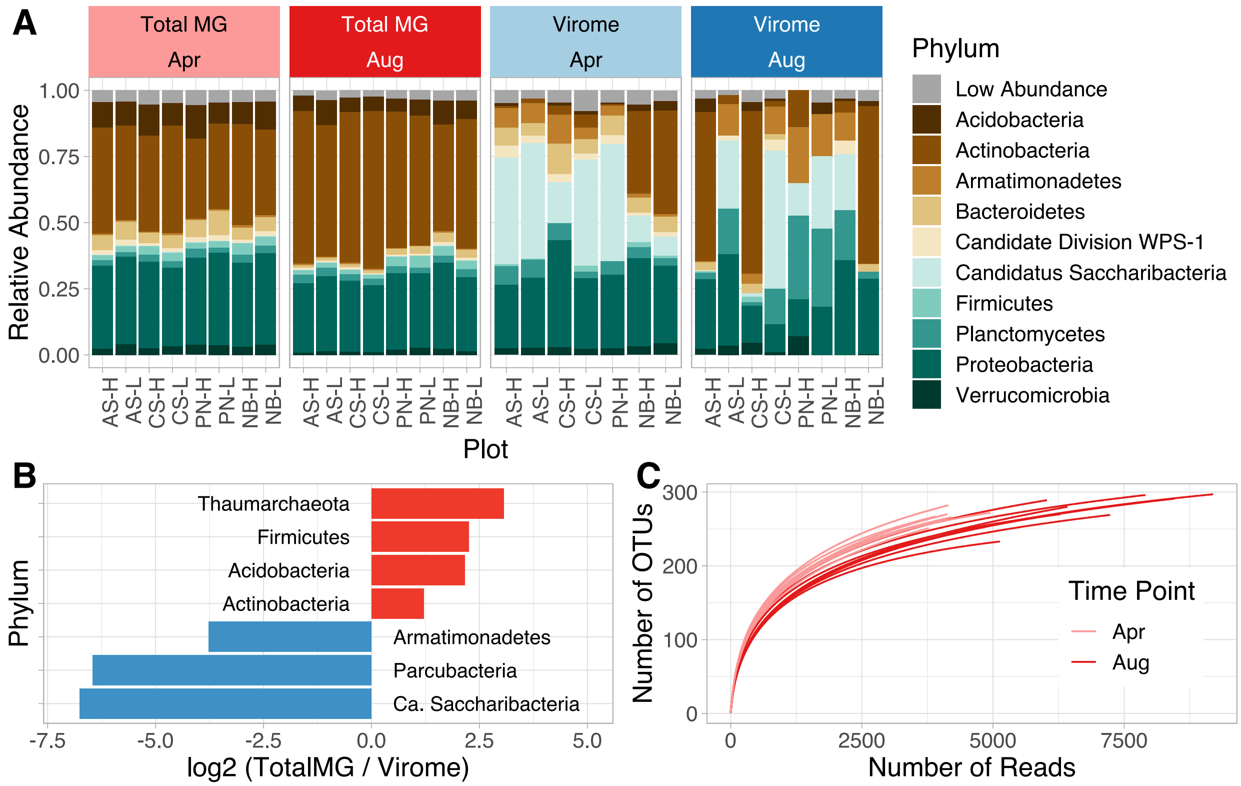

Supplementary Figure 2

(A) Relative abundances of microbial phyla in each library, based on recovered 16S rRNA gene fragments with an assigned taxonomy. Only the top 10 most abundant phyla are shown. “Low abundance” represents the summed relative abundances of all OTUs not assigned to the 10 most abundant phyla. (B) Microbial phyla with significantly different relative abundances between total metagenomes (MGs) and viromes (adjusted $P < 0.05$; paired Wilcoxon test). Red and blue bars indicate phyla significantly enriched in total metagenomes and viromes, respectively. Higher values along the x-axis indicate more significant enrichment. (C) Rarefaction curves of 16S rRNA gene OTU profiles derived from total metagenomes. Each line represents one metagenome.

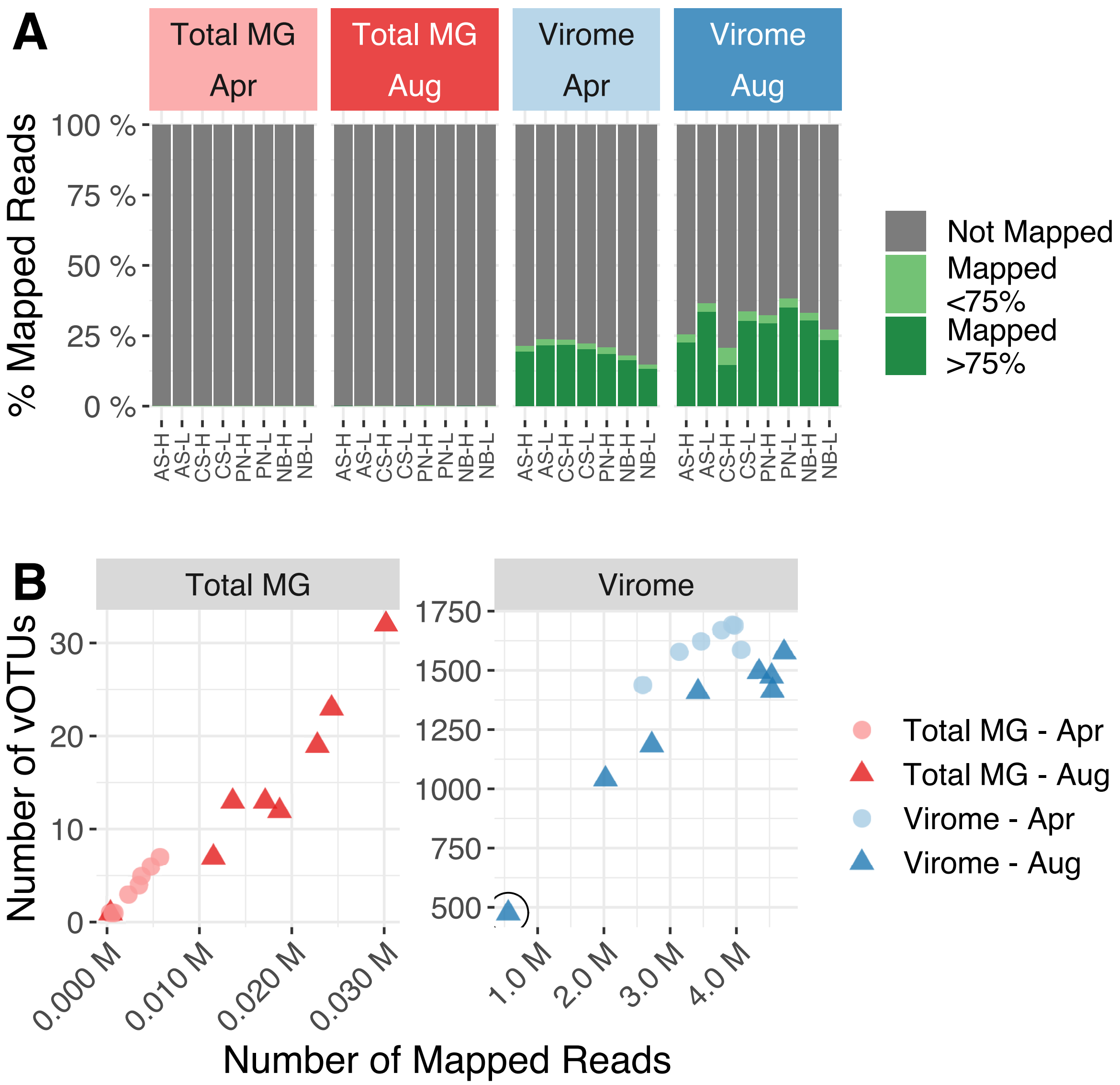

Supplementary Figure 3

(A) Percent of high-quality reads mapped to the set of vOTU contigs identified in our dataset. Green colors highlight read mapping below (light green) or above (dark green) the threshold of 75% coverage over the length of the vOTU sequence required for detection. (B) Distribution of the total number of reads mapped to vOTUs (x-axis) and the total number of vOTUs detected in each sample (y-axis). Note different y-axis maxima between graphs. The circled virome sample performed suboptimally (>2 standard deviations below the mean number of mapped reads and mean richness across viromes) and was therefore discarded for downstream compositional analyses. Total MG = total metagenome.

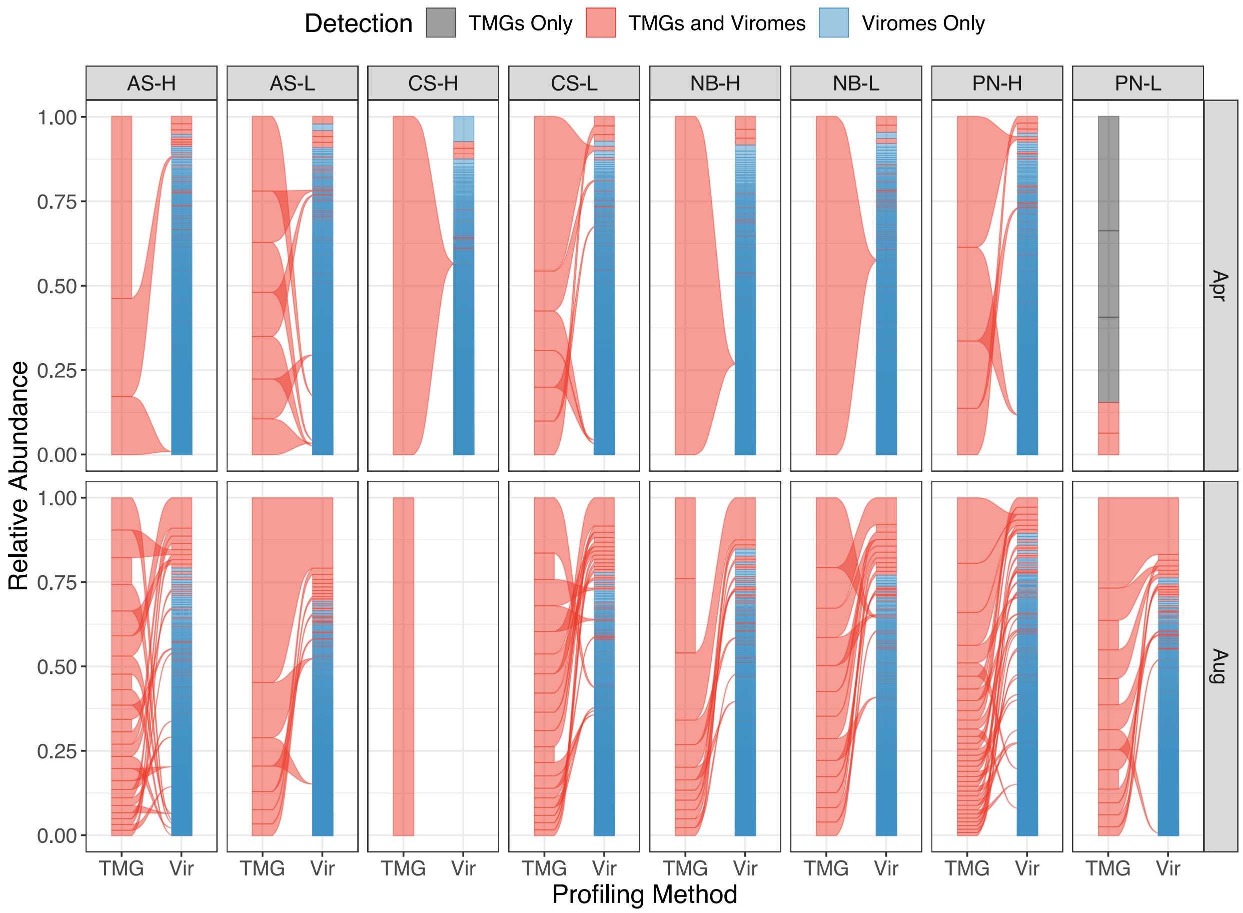

Supplementary Figure 4

Ranked relative abundances of individual vOTUs across profiling methods. Facets display individual sets of paired total metagenomes (TMGs) and viromes (Vir) derived from the same soil sample. The position of each vOTU along the y-axis indicates its rank within a profile, and a link between two profiles indicates that the particular vOTU was found in both the total metagenome and the virome from the same sample. Color denotes the detection category for each vOTU, considering all viromes and total metagenomes (as in the Euler diagram in Figure 2C): blue indicates vOTUs exclusively detected in viromes, gray indicates vOTUs exclusively detected in total metagenomes, and red indicates vOTUs found in both datasets.

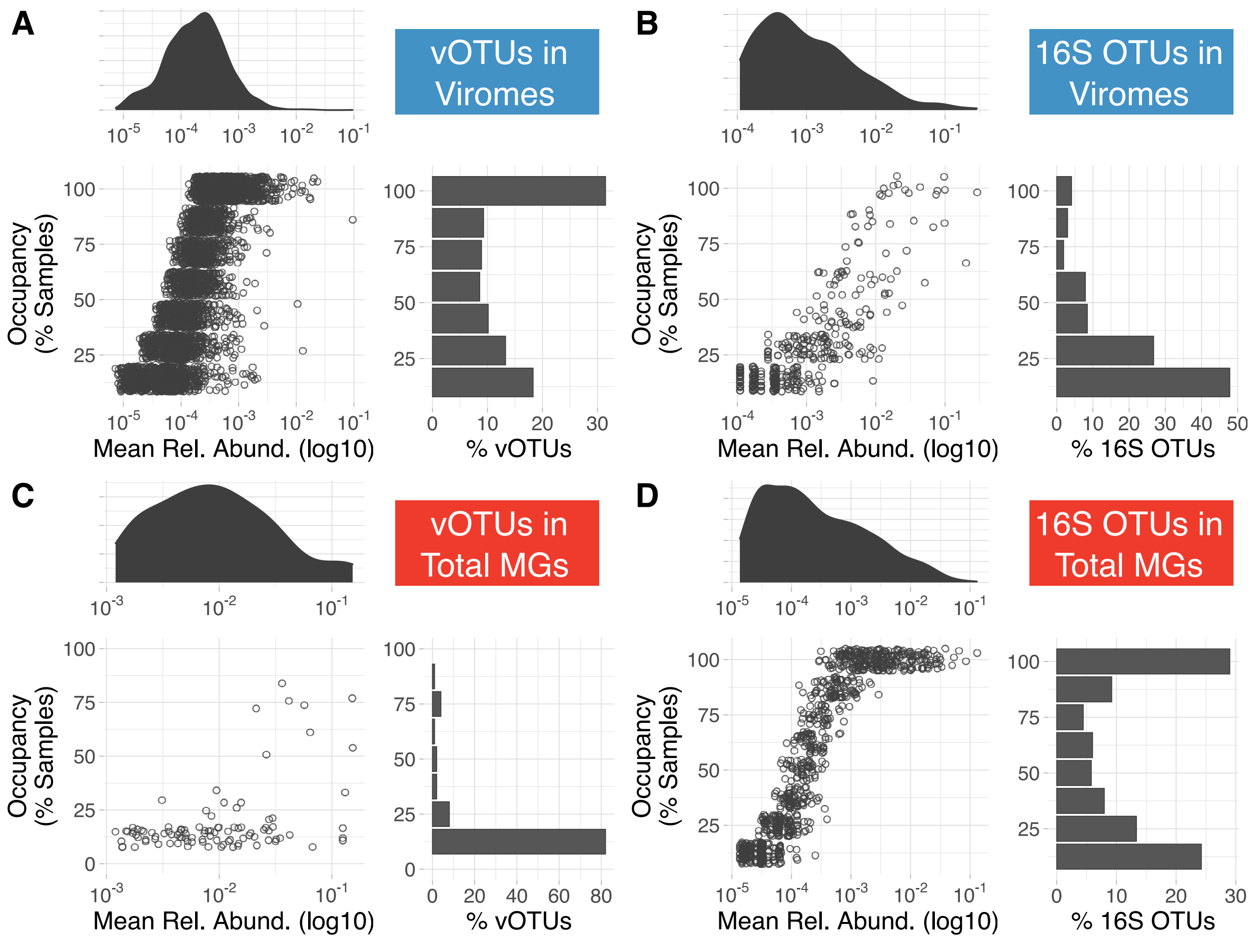

Supplementary Figure 5

Abundance-occupancy curves of vOTUs in viromes (A), 16S OTUs in viromes (B), vOTUs in total metagenomes (MGs) (C), and 16S rRNA gene OTUs in total metagenomes (D). In all panels, the bottom left scatter plots represent the mean relative abundance (x-axis) and occupancy (percent of samples in which a given vOTU was detected, y-axis) that individual vOTUs or 16S OTUs displayed within a collection time point (April or August). Thus, vOTUs or 16S OTUs detected in both time points are represented twice. The top left density curves show the distribution of relative abundances for all vOTUs or 16S OTUs. The bottom right bar plots display the percent of vOTUs or 16S OTUs (x-axis) found at each occupancy level (y-axis).

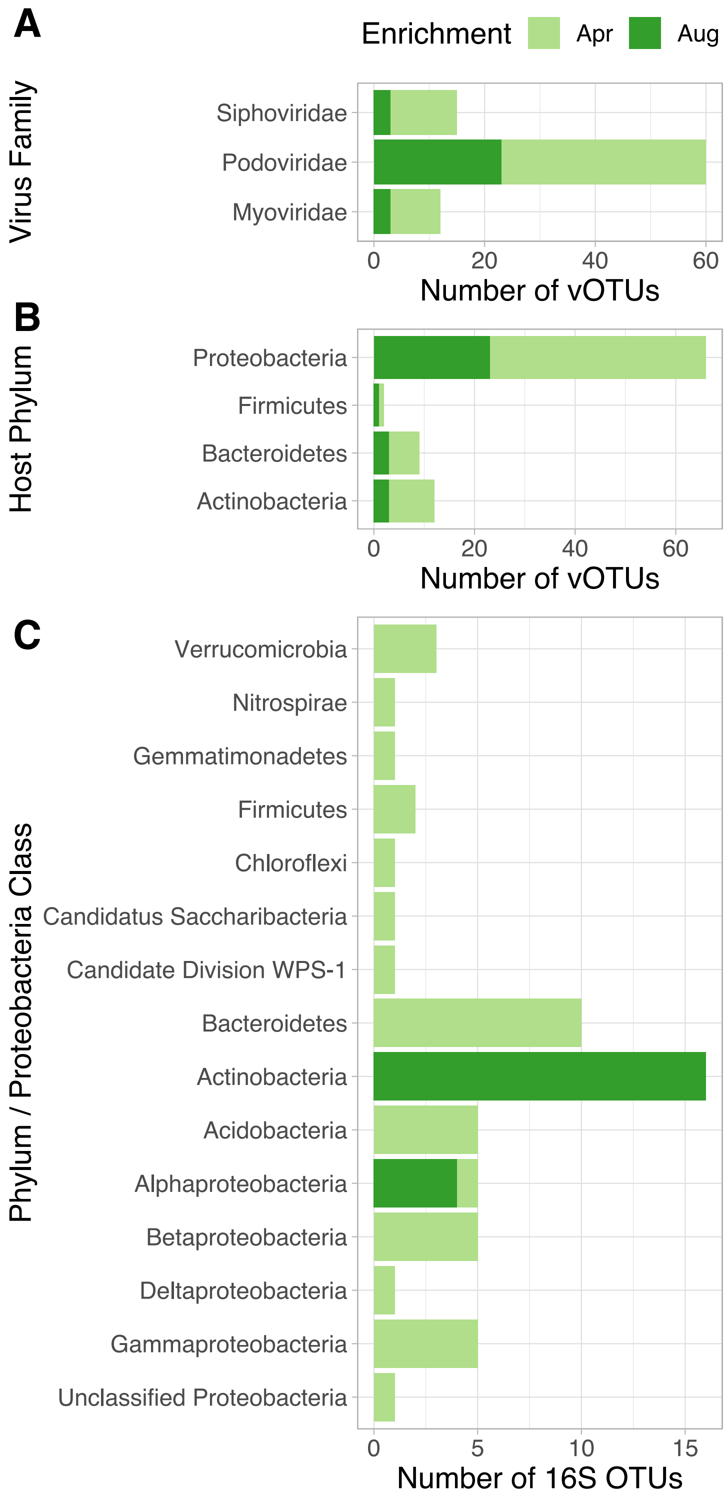

Supplementary Figure 6

(A) Virus family and (B) predicted host phylum of vOTUs that were significantly enriched according to collection time point and that could be taxonomically classified (n = 92 vOTUs) (**Fig 3**). (C) Phylum or Proteobacteria class of the 16S rRNA gene OTUs that were significantly differentially abundant between collection time points. In all plots, color indicates the collection time point in which vOTUs or 16S rRNA gene OTUs were enriched.

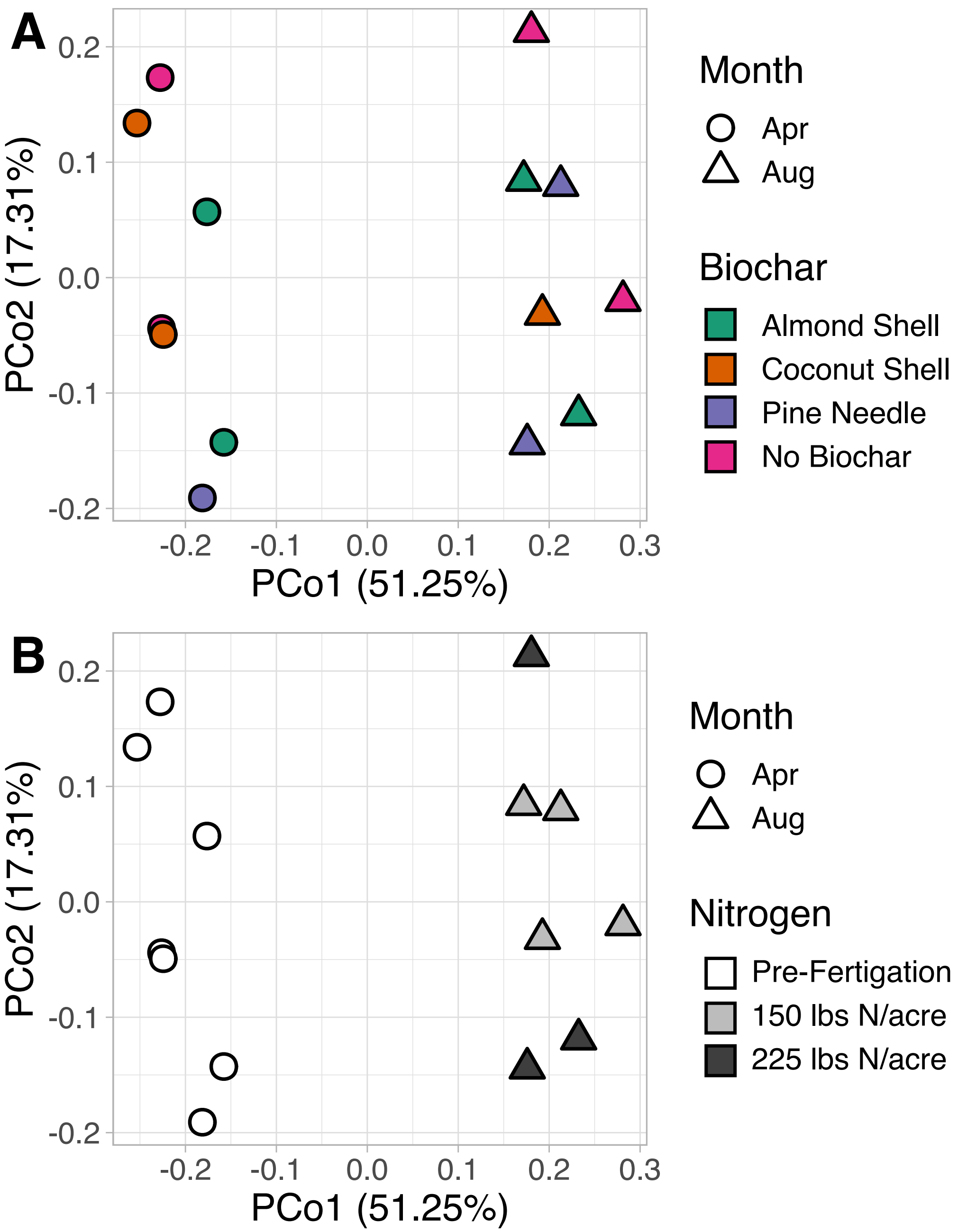

Supplementary Figure 7.
Unconstrained analysis of principal coordinates based on vOTU Bray-Curtis dissimilarities calculated on Hellinger-transformed relative abundances across virome samples. Shapes represent the collection time point, and colors indicate (A) biochar treatment or (B) nitrogen fertilization treatment (note that April samples were collected before any nitrogen was added to the field)

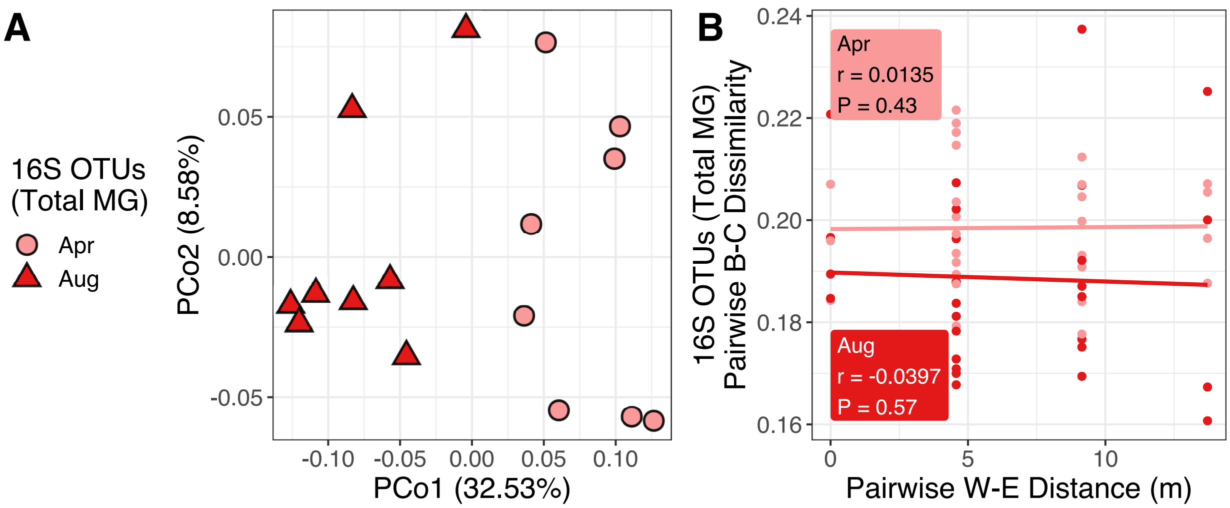

Supplementary Figure 8.

(A) Unconstrained analyses of principal coordinates based on Bray-Curtis (B-C) dissimilarities calculated on Hellinger-transformed relative abundances across 16S rRNA gene OTU profiles derived from total metagenomes (MGs). (B) Correlation between spatial distance across the west-east axis (in meters between plots) and Bray-Curtis dissimilarities calculated across 16S rRNA gene OTU profiles derived from total metagenomes. Inset values display the Mantel r statistic and associated P-value. Bray-Curtis dissimilarities were calculated on Hellinger-transformed relative abundances.

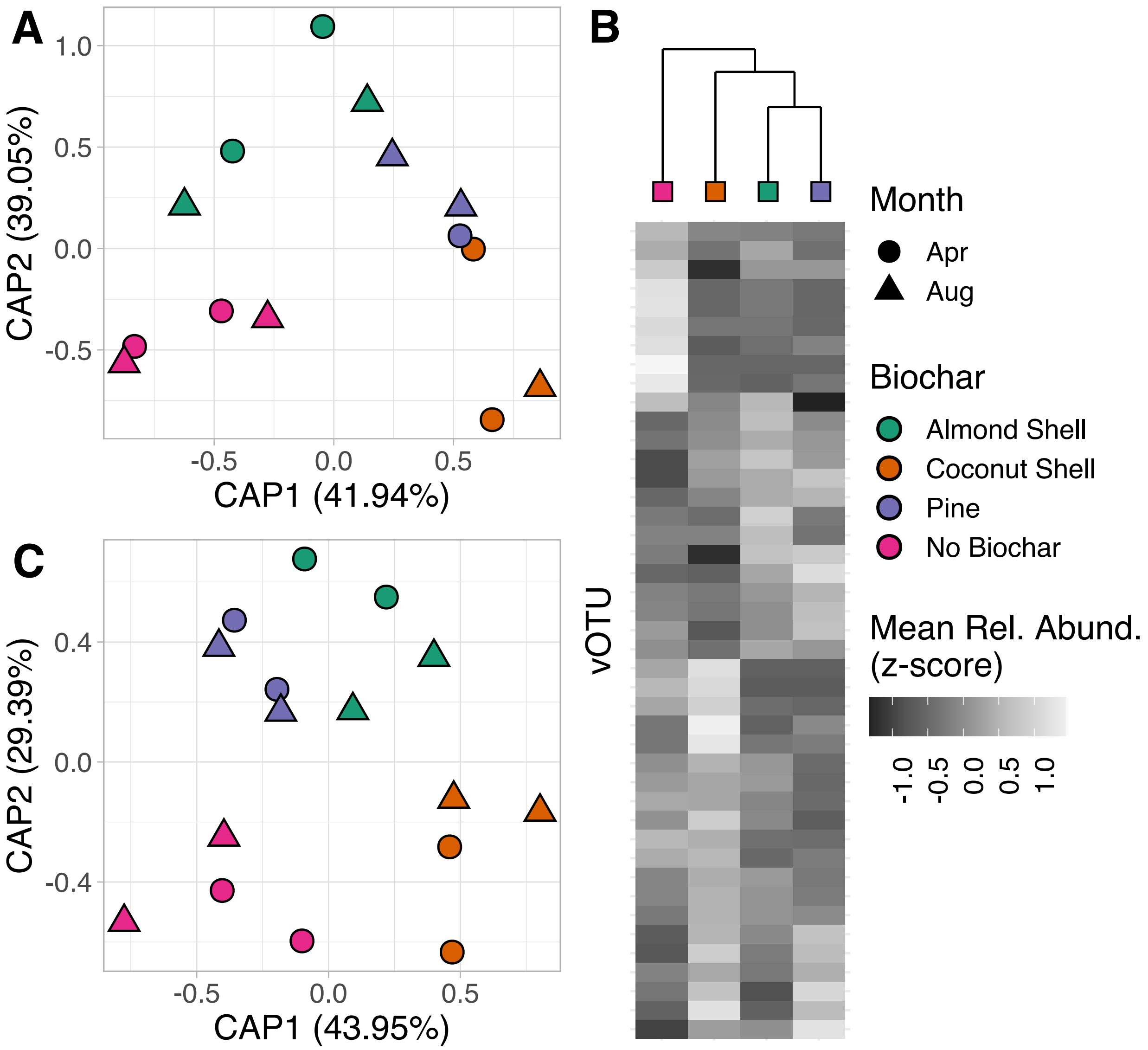

Supplementary Figure 9.
(A) Partial canonical analysis of principal coordinates (CAP) performed on Bray-Curtis dissimilarities calculated on vOTU profiles from viromes. The effects of collection time point and W-E position were removed. Colors indicate biochar treatment, and shape indicates sampling time point (legends are to the right of panel B). (B) Hierarchical clustering of biochar treatments based on the relative abundances of vOTUs significantly affected by biochar amendments. The heatmap shows the mean relative abundance (z-transformed) of each vOTU (rows) across biochar treatments (columns). (C) Partial CAP performed on Bray-Curtis dissimilarities calculated on 16S rRNA gene OTU profiles from total metagenomes. For A and B, Bray-Curtis dissimilarities were calculated on Hellinger-transformed relative abundances. The effect of collection time point was removed.

Supplementary Table Legends

Supplementary Table 1
Properties of biochar amended to agricultural field

Supplementary Table 2
Sequencing depths (before and after quality filtering) and sample metadata for all libraries reported in this study.

Supplementary Table 3
Viral cluster assignment for RefSeq genomes and vOTU contigs

Supplementary Table 4
Permutational multivariate analyses of variance testing the effect of individual variables on community composition. Analyses were performed on Bray-Curtis dissimilarities calculated on Hellinger-transformed relative abundances across vOTU profiles in viromes and 16S rRNA gene OTU profiles in total metagenomes (Total MG). The effect of nitrogen concentration was only tested in the August subset of samples, after the fertigation had occurred. The last column indicates the formula used to run the test.

Supplementary Table 5
Permutational multivariate analyses of variance testing the effect of biochar or nitrogen treatments while controlling for the variation due to collection time point and/or W-E spatial gradient. Analyses were performed on Bray-Curtis dissimilarities calculated on Hellinger-transformed relative abundances across vOTU profiles in viromes and 16S rRNA gene OTU profiles in total metagenomes (Total MG). The effect of nitrogen concentration was only tested in the August subset of samples, after the fertigation had occurred. The last column indicates the formula used to run the test.

Supplementary Table 6
Set of vOTUs differentially abundant across collection time points (Wald test, adjusted P-val < 0.05). The first 7 columns are the default output from DESeq2. Column “VC” indicates the viral cluster assigned to each vOTU by vConTACT2.

Supplementary Table 7
Set of 16S rRNA gene OTUs differentially abundant across collection time points (Wald test, adjusted P-val < 0.05). The first 7 columns are the default output from DESeq2.

Supplementary Table 8
ANOVA test of the effects of collection time point, biochar, nitrogen amendment concentration, W-E position, and S-N position on the measured chemical properties of soil. The effect of nitrogen amendment concentration was only tested for the August samples.

Supplementary Table 9
Set of vOTUs significantly affected by plot position along the west-east axis of the sampled field (Wald test, adjusted P-val < 0.05). The first 7 columns are the default output from DESeq2. Column “VC” indicates the viral cluster assigned to each vOTU by vConTACT2.

Supplementary Table 10

Set of vOTUs differentially abundant across biochar treatments (likelihood ratio test, adjusted P-val < 0.05). Column “VC” indicates the viral cluster assigned to each vOTU by vConTACT2.
